# Interface solvation regulation stabilizing the Zn metal anode in aqueous Zn batteries†

**DOI:** 10.1039/d3sc01831h

**Published:** 2023-07-11

**Authors:** Kuo Wang, Tong Qiu, Lu Lin, Fangming Liu, Jiaqi Zhu, Xiao-Xia Liu, Xiaoqi Sun

**Affiliations:** a Department of Chemistry, Northeastern University Shenyang 110819 China sunxiaoqi@mail.neu.edu.cn

## Abstract

The Zn metal anode experiences dendritic growth and side reactions in aqueous zinc batteries. The regulation of the interface environment would provide efficient modification without largely affecting the aqueous nature of bulk electrolytes. Herein, we show that the ethylene carbonate (EC) additive is able to adsorb on the Zn surface from the ZnSO_4_ electrolyte. Together with the higher dielectric constant of EC than water, Zn^2+^ preferentially forms EC-rich solvation structures at the interface even with a low overall EC content of 4%. An inorganic–organic solid-electrolyte interface (SEI) is also generated. Thanks to the increased energy levels of the lowest unoccupied molecular orbital of EC-rich solvation structures and the stable SEI, side reactions are suppressed and the Zn^2+^ transference number increases to allow uniform Zn growth. As a result, the cycle life of Zn stripping/plating in symmetric Zn cells extends from 108 h to 1800 h after the addition of 4% EC. Stable cycling for 180 h is realized with 35% depth of discharge in the 4% EC electrolyte, superior to the initial cell failure with EC-free electrolyte. The capacity retention of the Zn//V_6_O_13_·H_2_O full cell with N/P = 1.3 also increases from 51.1% to 80.5% after 500 cycles with the help of EC.

## Abstract 2

The ethylene carbonate additive regulates Zn anode behavior, enhancing the capacity retention of the full cell (N/P = 1.3) from 51.1% to 80.5%.

## Graphical abstract

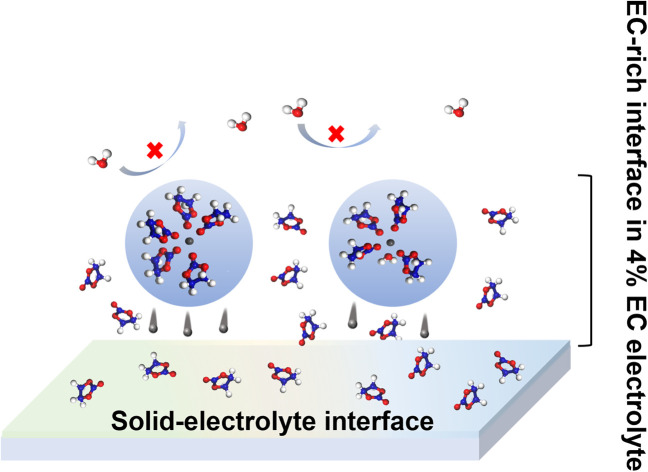

## Introduction

Rechargeable aqueous zinc batteries have been widely studied as an advanced energy storage system due to their low toxicity and low cost.^1–6^ The zinc metal anode provides high theoretical capacity (820 mA h g^−1^) and low redox potential (−0.76 V *vs.* SHE). However, it also experiences dendritic growth and side reactions in aqueous batteries, which hinder further developments.^7–20^ The dendrite formation results from the inhomogeneous nucleation of Zn, followed by the preferential crystal growth on existing nuclei to minimize the surface energy. Side reactions, mainly the hydrogen evolution reaction (HER), are attributed to the thermodynamic instability of Zn metal in a mild acidic environment and competitive reduction of Zn^2+^ and H^+^ during the Zn deposition process.

It has been demonstrated that Zn^2+^ solvation structures in electrolytes are essential in determining the deposition/dissolution behavior and any possible side reactions. Accordingly, a few strategies have been proposed to regulate the solvation structures. Dimethyl sulfoxide,^21^ acetonitrile,^22,23^ methanol^24^ and a few carbonates^25–27^ were introduced in electrolytes, respectively. They can replace the water in the Zn^2+^ solvation shells, which helped guide uniform Zn^2+^ deposition and inhibit the HER. These organic additives also generate solid-electrolyte interface (SEI) on the Zn electrode to further solve the related problems. Nevertheless, in order to change the Zn^2+^ solvation structures in bulk electrolytes, additives need to be introduced stoichiometrically with respect to Zn^2+^ to enter all solvation shells. The addition of excessive additives would sacrifice the low toxicity and low cost advantages of aqueous electrolytes.

**Fig. 1:**
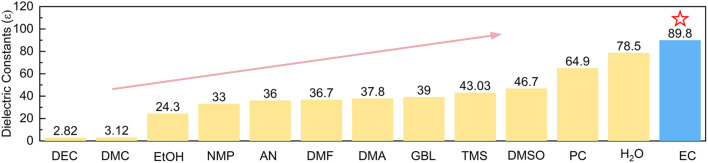
Dielectric constants of EC and other common solvents including H_2_O.

Since Zn deposition/dissolution or side reactions take place at the interface between the Zn electrode and electrolyte, it is more efficient to regulate the interface environment in order to modify the electrochemical performance of the Zn electrode. If additives are able to accumulate at the inner Helmholtz layer of the Zn electrode and present a high tendency of coordination with Zn^2+^, it would effectively change Zn^2+^ solvation structures at the interface while bulk electrolytes remain aqueous. Polar solvents, in particular, are able to weaken the interactions between anions and cations. They would further enter Zn^2+^ solvation shells provided that stable solvation structures can be formed. We herein introduce ethylene carbonate (EC), which possesses the dielectric constant (89.8*ε*) higher than that of most reported organic solvents (Fig. 1) and corresponds to larger polarity, as the additive to the typical 3 m ZnSO_4_ electrolyte (units in mol kg^−1^, around 2.2 mol L^−1^). Theoretical calculations and experimental analysis confirm the effective adsorption of EC on the Zn surface, which preferentially generates ZnEC_5_H_2_O^2+^ and ZnEC_6_^2+^ solvation structures at the interface. They present higher energy levels of the lowest unoccupied molecular orbital (LUMO) than Zn(H_2_O)_6_^2+^, corresponding to higher HER resistance. EC molecules also induce a stable inorganic–organic SEI layer on Zn. It further suppresses side reactions, and the Zn^2+^ transference number increases to ensure uniform Zn deposition. Thanks to the above effects, the coulombic efficiencies of Zn plating/stripping reach 99.4% for 600 cycles in the 4% EC electrolyte, and the cycle life of symmetric Zn cells extends from 108 h to 1800 h at 1 mA cm^−2^ and 1 mA h cm^−2^ after the addition of EC. Importantly, Zn stripping/plating with 35% depth of discharge (DOD) achieves a cycle life of 180 h in 4% EC, which is superior to the initial failure of the cell with EC-free electrolyte. The capacity retentions of Zn//V_6_O_13_·H_2_O full batteries with N/P = 1.3 (based on theoretical capacities) also increase from 51.1% to 80.5% after 500 cycles with the help of EC.

## Results and discussion

**Fig. 2:**
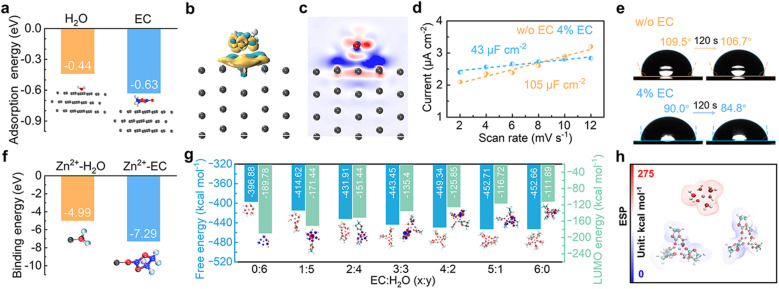
(a) Adsorption energies of solvent molecules (H_2_O or EC) on the Zn (100) facet. (b) Charge density difference and (c) sliced 2D contour map reflecting the interactions between EC and Zn at the interface. (d) Linear fits to calculate the EDLC of Zn in different electrolytes. (e) Contact angle measurements of Zn foil in different solutions before and after 120 s. (f) Binding energies between Zn^2+^ and solvent molecules (H_2_O or EC). (g) Free energies and LUMO energy levels of ZnEC_*x*_(H_2_O)_*y*_^2+^ (*x* + *y* = 6) complexes. (h) Electrostatic surface potentials of Zn(H_2_O)_6_^2+^, ZnEC_5_H_2_O^2+^ and ZnEC_6_^2+^.

The interface environment between the Zn electrode and the electrolyte is essential in determining Zn deposition behavior and possible side reactions. The influence of EC on the interface is thus first studied. The adsorption energies of EC and H_2_O molecules on the Zn (100) crystal plane are calculated (Fig. 2a). EC exhibits larger adsorption energy of −0.63 eV in comparison to −0.44 eV for H_2_O, indicating that EC molecules preferentially adsorb on the Zn surface in the mixed solution containing EC and water. Fig. 2b and c show the charge density difference and 2D contour map of electron density statistics at the interface between EC and Zn. It demonstrates the transfer of electron density from EC to the surface of Zn, confirming the effective interactions. The interface environment is further studied by calculating the electrochemical double layer capacitance (EDLC) of the Zn electrode in the 3 m ZnSO_4_ aqueous electrolyte and after adding 4% EC (labeled as w/o EC and 4% EC, respectively) with cyclic voltammetry (CV) in the non-Faraday range (Fig. S1†). According to linear fits (Fig. 2d), the Zn electrode exhibits the EDLC of 105 μF cm^−2^ in 3 m ZnSO_4_, which decreases to 43 μF cm^−2^ in 4% EC. It corresponds to the increase of Stern layer distance, resulting from the replacement of water molecules by larger sized EC in the inner Helmholtz layer of the Zn electrode.^27,28^Fig. 2e shows the contact angles of 3 m ZnSO_4_ and 4% EC solutions on Zn foil. The angles of 3 m ZnSO_4_ change from 109.5° to 106.7° after 120 s, while those of 4% EC reduce from 90° to 84.8°. The smaller angles of the latter are attributed to the adsorption of EC molecules on Zn. The above analysis demonstrates that the interface of the Zn electrode in the 4% EC electrolyte is EC rich locally. It would generate different solvation structures for Zn^2+^ at the interface from the bulk electrolyte.

The interactions between Zn^2+^ and EC or H_2_O molecules are studied by density functional theory (DFT) calculations. It results in a much larger binding energy of Zn^2+^–EC than Zn^2+^–H_2_O (−7.29 eV *vs.* −4.99 eV, Fig. 2f), suggesting the favorable coordination of Zn^2+^ with EC. The free energies of ZnEC_*x*_(H_2_O)_*y*_^2+^ (*x* + *y* = 6) complexes with different *x* and *y* values are calculated, and results are summarized in Fig. 2g. SO_4_^2−^ is not included for the calculation due to its low participation in the inner solvation shells of Zn^2+^ as confirmed by molecular dynamics (MD) simulation and Raman analysis (Fig. S2†). With the substitution of solvated water by EC, the free energy decreases from −396.88 kcal mol^−1^ for Zn(H_2_O)_6_^2+^ to −452.71 kcal mol^−1^ and −452.66 kcal mol^−1^ for ZnEC_5_H_2_O^2+^ and ZnEC_6_^2+^, respectively, which corresponds to increased stability. In accordance, the electrostatic surface potentials of Zn(H_2_O)_6_^2+^, ZnEC_5_H_2_O^2+^ and ZnEC_6_^2+^ show that the solvation structures are more stable with EC molecules replacing water in the coordination shell (Fig. 2h).^29^ The results demonstrate that ZnEC_5_H_2_O^2+^ and ZnEC_6_^2+^ are the favorable species at the EC-rich interface of the Zn electrode, despite the low overall EC concentration in the bulk electrolyte.

According to previous studies, the solvated water around Zn^2+^ is mainly responsible for the HER side reaction at the Zn electrode.^30^ The LUMO energy levels of ZnEC_*x*_(H_2_O)_*y*_^2+^ species are calculated. As shown in Fig. 2g, the LUMO level increases with more EC replacing water in the solvation shell, corresponding to more difficult reduction. Therefore, the formation of ZnEC_5_H_2_O^2+^ and ZnEC_6_^2+^ structures instead of Zn(H_2_O)_6_^2+^ at the interface helps to suppress HER side reactions. Meanwhile, MD simulation and spectroscopy analysis also suggest the formation of hydrogen bonds among EC and water molecules (Fig. S3†). It further helps to reduce water activity and inhibit the HER.

**Fig. 3:**
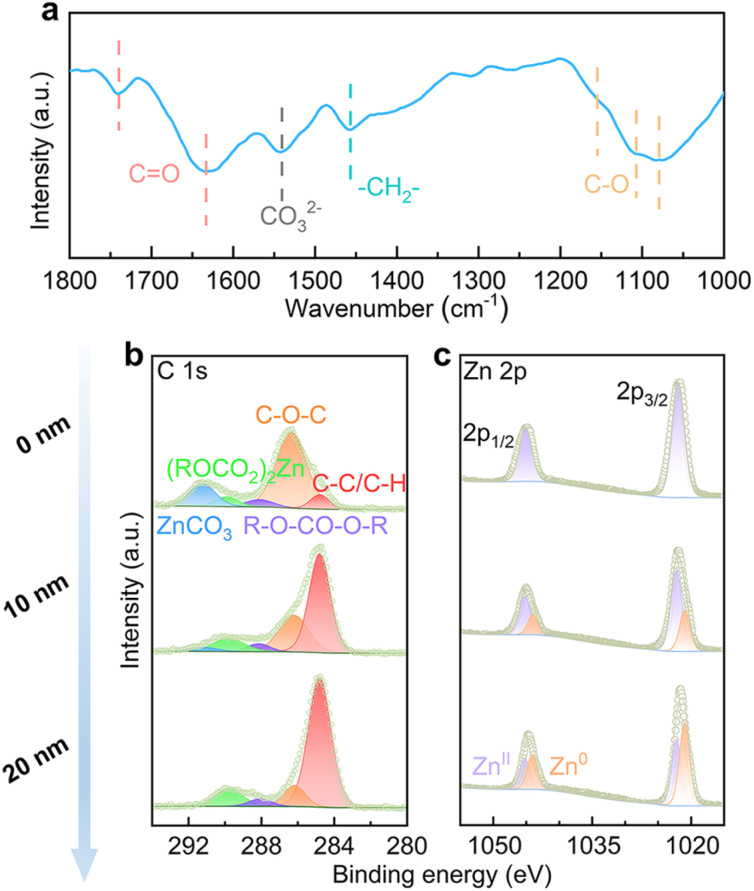
(a) FT-IR, (b) C 1s and (c) Zn 2p XPS with different sputtering depths by Ar^+^ of the Zn electrode after 25 cycles in the 4% EC electrolyte.

The EC species at the interface, both the ones solvated with Zn^2+^ and free molecules, may generate SEI on the Zn electrode over electrochemical cycling. This is studied by Fourier transform infrared (FT-IR) spectroscopy with the reflection mode on a Zn electrode after 25 stripping/plating cycles (2 mA cm^−2^, 2 mA h cm^−2^). As shown in Fig. 3a, the stretching vibrations of C–O at 1079 cm^−1^, 1107 cm^−1^ and 1155 cm^−1^ are attributed to ether from PEO-type polymers, alkyl carbonate salts ((ROCO_2_)_2_Zn) and alkyl carbonate (R–O–CO–O–R), respectively.^31^ The bending vibration of –CH_2_– shows up at 1457 cm^−1^, and the stretching vibration of carbonate from ZnCO_3_ shows up at 1540 cm^−1^.^32^ The stretching vibrations of C=O from alkyl carbonate salts and alkyl carbonate appear at 1633 cm^−1^ and 1740 cm^−1^, respectively.^33^ The above species originate from EC decomposition on the Zn electrode.

The SEI composition is further studied by X-ray photoelectron spectroscopy (XPS) with different etching depths. In the C 1s spectrum of an un-etched electrode (Fig. 3b), signals from C–O–C, R–O–CO–O–R, (ROCO_2_)_2_Zn and ZnCO_3_ are noted.^34^ With the increase of etching depth, ZnCO_3_ disappears and the intensities of other components decrease in comparison to adventitious carbon. In the Zn 2p spectra (Fig. 3c), the un-etched electrode shows mainly the Zn^II^ signal, which is attributed to (ROCO_2_)_2_Zn and ZnCO_3_. The Zn^II^ signal decreases and Zn^0^ increases upon etching, as a result of the removal of coverage on Zn metal. Overall, the analysis suggests that the inner SEI contains PEO-type polymers, alkyl carbonate and (ROCO_2_)_2_Zn, and additional ZnCO_3_ is found on the top surface.

**Fig. 4:**
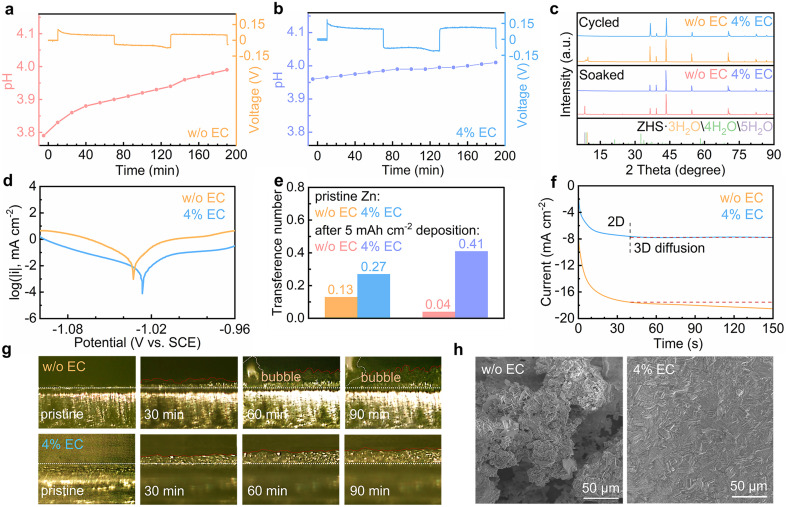
*In situ* pH measurements of (a) 3 m ZnSO_4_ and (b) 4% EC electrolytes during the Zn stripping/plating process. (c) XRD patterns of Zn soaked for 24 h and after 25 cycles, (d) Tafel plots of the Zn electrode, (e) Zn^2+^ transference numbers with the pristine Zn electrode and after 5 mA h cm^−2^ deposition, (f) CA curves at −150 mV *vs.* Zn constant potential, (g) *in situ* optical microscopy images during Zn deposition, and (h) SEM images of the Zn electrode after 25 cycles in ZnSO_4_ and 4% EC electrolytes.

The effect of EC interface regulation on the stability of the Zn electrode is studied. The HER behaviors of Zn in the two solutions are explored by *in situ* pH measurements (Fig. 4a and b). Symmetrical Zn//Zn cells are assembled, and the evolutions of electrolyte pH are monitored during the repeated galvanostatic Zn stripping/plating process. In the 3 m ZnSO_4_ electrolyte, the pH increases from 3.79 to 3.83 during the initial rest period. It results from the chemical displacement reaction between the proton and Zn. When the current turns on, the pH values keep increasing during both stripping and plating processes. It results from the continuous chemical displacement reaction as well as the electrochemical HER process. In the 4% EC electrolyte, the initial pH is slightly higher than the neat solution. The pH change is below 0.05 after the rest period as well as stripping/plating processes. It demonstrates the effective suppression of the HER with the help of EC. Fig. 4c shows the X-ray diffraction (XRD) patterns of Zn electrodes after soaking for 24 h in the two electrolytes or after 25 galvanostatic stripping/plating cycles (2 mA cm^−2^, 2 mA h cm^−2^). The Zn electrodes from 3 m ZnSO_4_ present apparent diffractions from zinc basic salts, as a result of local pH increase from chemical and electrochemical HER processes. In contrast, no such peaks are observed from the 4% EC electrolyte thanks to the inhibited HER. Fig. S4† shows the electrochemical window of the two electrolytes. The extended window on both sides of 4% EC confirms the enhanced HER resistivity as well as suppressed oxygen evolution reaction (OER) by EC. Fig. 4d shows the Tafel plots. EC enables a decrease of corrosion current from 17.47 μA cm^−2^ to 3.27 μA cm^−2^ and an increase of corrosion potential from −1.033 V to −1.026 V (*vs.* SCE). This inhibited corrosion is attributed to the replacement of water by EC in the solvation shell of Zn^2+^ at the interface, as well as the prevented contact between Zn and the electrolyte by the SEI.

The effect of EC on Zn^2+^ transport is evaluated by calculating the transference numbers of the Zn^2+^ cation (Fig. S5† and 4e).^35,36^ The Zn^2+^ transference numbers in ZnSO_4_ without and with EC are 0.13 and 0.27 with the pristine Zn electrode. The higher value of the latter is attributed to the interactions between Zn^2+^ and EC at the interface. Deposition processes are then carried out on the Zn electrode in the two electrolytes for 5 mA h cm^−2^ capacity, which at the same time generates the stable SEI or side products on the Zn surface. The transference number in the 4% EC electrolyte increases to 0.41. It suggests that the SEI helps to further enhance Zn^2+^ transport. In ZnSO_4_, in contrast, the transference number decreases to 0.04. This hindered Zn^2+^ transport is attributed to the side products of zinc basic salts formed in the EC-free electrolyte. The above experiments confirm that the SEI on Zn helps to facilitate Zn^2+^ transport. It would reduce the cation gradient and regulate Zn deposition behaviors. Chronoamperometry (CA) is carried out to study the deposition process (Fig. 4f). With the constant potential of −150 mV *vs.* Zn, the deposition current density continuously increases for more than 150 s in the 3 m ZnSO_4_ electrolyte, corresponding to the formation of uneven Zn deposits. In comparison, the current density exhibits negligible change after the initial 40 s in the 4% EC electrolyte. It results from the inhibition of lateral diffusion of Zn on the surface, which ensures uniform Zn deposition.^37,38^

*In situ* optical microscopy is applied to monitor the Zn^2+^ deposition behavior. Fig. 4g shows images of the Zn interface in the two electrolytes. In 3 m ZnSO_4_, irregular deposits appear with the increase of deposition time, and bubbles are also generated from the HER. In contrast, Zn deposited from 4% EC grows uniformly on the surface. The smaller thickness than in ZnSO_4_ suggests denser deposition. No corrosion behavior is noted, either. Fig. 4h shows the scanning electron microscopy (SEM) images of the Zn electrode after 25 stripping/plating cycles (2 mA cm^−2^, 2 mA h cm^−2^). The deposits aggregate on the surface of Zn from the 3 m ZnSO_4_ electrolyte, whereas a smooth and uniform Zn surface is obtained from 4% EC.

**Fig. 5:**
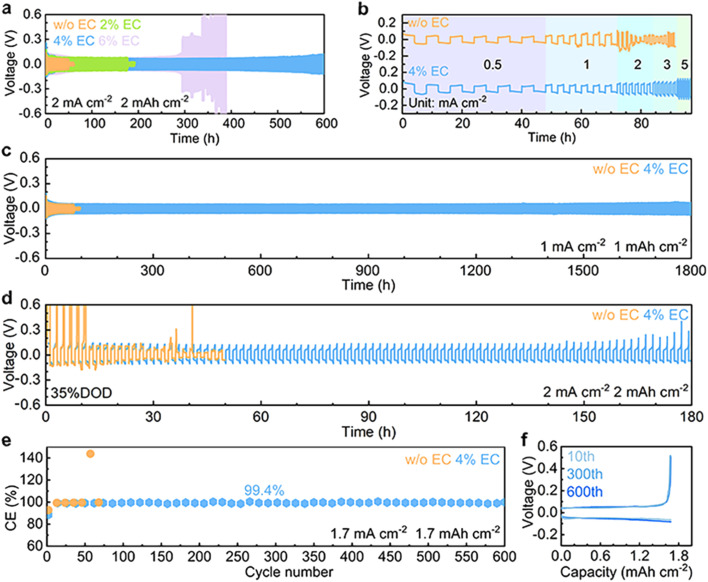
(a) Long-term cycling at 2 mA cm^−2^ and 2 mA h cm^−2^ in 3 m ZnSO_4_ electrolyte and with different concentrations of the EC additive. (b) Rate performance, (c) long-term cycling at 1 mA cm^−2^ and (d) cycling stability with 35% DOD (thin foil) of Zn stripping/plating in Zn//Zn symmetric cells with 3 m ZnSO_4_ and 4% EC electrolytes. (e) Coulombic efficiencies of Zn plating/stripping in Zn//Cu cells in the two electrolytes and (f) voltage curves in 4% EC.

The electrochemical performance of Zn stripping/plating in different electrolytes is evaluated in symmetric Zn//Zn cells. The 4% EC additive is confirmed to be optimal by the cycling tests at 2 mA cm^−2^ and 2 mA h cm^−2^ (Fig. 5a), and further comparisons are made between EC-free and 4% EC electrolytes. Fig. 5b shows the voltage curves at current densities from 0.5 mA cm^−2^ to 5 mA cm^−2^ and a capacity of 2 mA h cm^−2^. In the 3 m ZnSO_4_ electrolyte, the cell short circuits at the current density of 2 mA cm^−2^. In contrast, the cell with 4% EC electrolyte functions properly at all current densities. Long-term cycling is carried out at 1 mA cm^−2^ and 1 mA h cm^−2^ (Fig. 5c). In ZnSO_4_, cell short-circuiting takes place at 108 h. The cycle life extends 16.7 times to 1800 h after EC addition. This performance is competitive with previous studies (Table S1†). Symmetric cells are further assembled with thin Zn electrodes (9.7 μm), and stripping/plating is carried out at 2 mA cm^−2^ current density and 2 mA h cm^−2^ capacity, which corresponds to 35% DOD (Fig. 5d). In 3 m ZnSO_4_, the voltage of the cell fluctuates greatly from the beginning. By contrast, the cell with 4% EC electrolyte exhibits 180 h stable cycles. The shorter lifetime obtained at 2 mA cm^−2^ and 2 mA h cm^−2^ in comparison to 1 mA cm^−2^ and 1 mA h cm^−2^ should be attributed to the higher depth of stripping/plating and potential sand behavior.^39^ In addition to EC, other carbonate additives including propylene carbonate (PC), diethyl carbonate (DEC), ethyl methyl carbonate (EMC) and dimethyl carbonate (DMC) also extend the cycle life of symmetric cells (Fig. S6†). Nevertheless, the best performance is obtained with EC. The Zn plating/stripping coulombic efficiencies are evaluated on Cu current collectors (Fig. 5e, f and S7†). The cell with the 3 m ZnSO_4_ electrolyte fails at the 72nd cycle, whereas the one with 4% EC delivers a stabilized CE of 99.4% for more than 600 cycles.

**Fig. 6:**
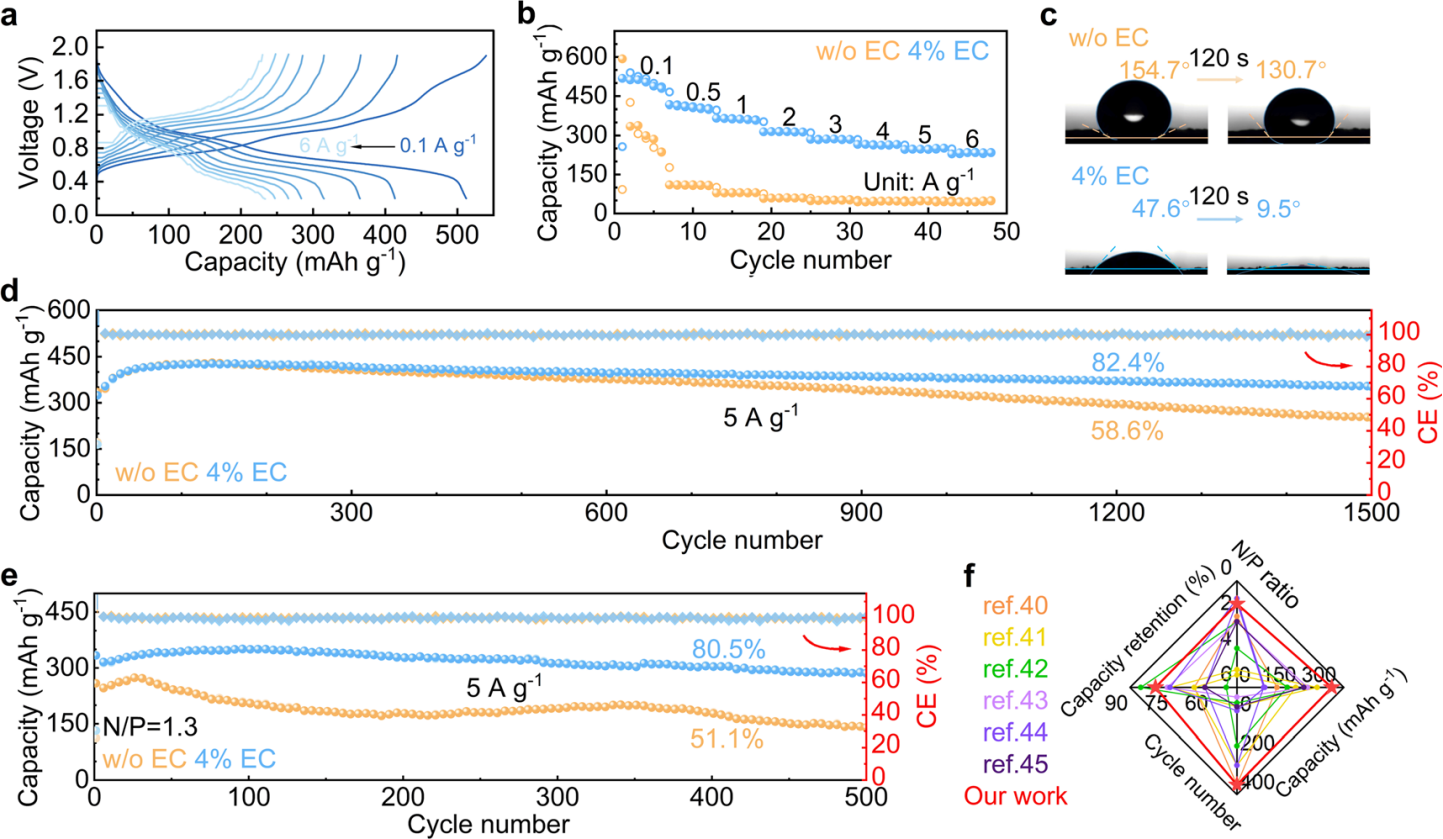
(a) Charge–discharge curves of the V_6_O_13_·H_2_O cathode in 4% EC and (b) rate performance in the two electrolytes. (c) Contact angles between the electrolytes and cathode before and after 120 s rest. Long-term cycling of V_6_O_13_·H_2_O at 5 A g^−1^ with (d) excess Zn anode and (e) limited Zn anode with N/P = 1.3. (f) Cycling performance comparison of full cells using limited Zn anodes with previous reports.

The 4% EC electrolyte is finally applied to a V_6_O_13_·H_2_O cathode in zinc cells. Galvanostatic charge and discharge are carried out at different current densities (Fig. 6a and b). In the 4% EC electrolyte, the cathode delivers a high capacity of 518 mA h g^−1^ at 0.1 A g^−1^, and 234 mA h g^−1^ capacity is retained with the increase of current density to 6 A g^−1^. By contrast, the cathode with 3 m ZnSO_4_ electrolyte exhibits much faster capacity decay at 0.1 A g^−1^ as well as poorer rate performance, and only 49 mA h g^−1^ capacity is left at 6 A g^−1^. Fig. 6c compares the contact angles of the two electrolytes on the V_6_O_13_·H_2_O cathode. The contact angles with ZnSO_4_ decrease from 154.7° to 130.7° after 120 s rest. By contrast, a much smaller angle 47.6° is obtained at the initial contact between 4% EC electrolyte and V_6_O_13_·H_2_O, which further decreases to 9.5° after 120 s. This greatly improved wettability by EC helps to reduce interfacial resistance, which ensures excellent rate capability.

Long-term cycling is carried out at 5 A g^−1^ (Fig. 6d and S8†). The V_6_O_13_·H_2_O cathode realizes 86.4% capacity retention after 1500 cycles in 4% EC, which is superior to 59.7% obtained in ZnSO_4_. The cycling stabilities are further evaluated in full cells with a limited anode of N/P = 1.3 (based on theoretical capacities). The full cell with 4% EC electrolyte exhibits 80.5% capacity retention after 500 cycles at 5 A g^−1^, with an average CE of 99.9%. In comparison, only 51.1% capacity retention is obtained in the 3 m ZnSO_4_ electrolyte (Fig. 6e and S9†). The cycling performance with the 4% EC electrolyte is also better than previously reported Zn full cells with limited anodes (Fig. 6f).^40–45^ The results confirm the promoted electrochemical performance by the EC additive for not only the Zn anode but also full cells.

## Conclusions

In summary, we show that the regulation of Zn^2+^ solvation structures at the electrode–electrolyte interface effectively enhances the electrochemical performance of the Zn anode. Specifically, the EC additive is introduced in the 3 m ZnSO_4_ aqueous electrolyte. Theoretical calculations and experimental analysis confirm that EC preferentially adsorbs on Zn over water. EC-rich solvation structures of Zn^2+^ are thus generated at the interface despite the low overall EC concentration in bulk electrolyte, and they possess higher HER resistance. An SEI layer composed of PEO-type polymers, alkyl carbonate, alkyl carbonate salts and ZnCO_3_ is also formed on Zn from EC decomposition. The above factors not only inhibit side reactions on Zn, but also increase the Zn^2+^ transference number, which ensures uniform Zn deposition. The Zn plating/stripping coulombic efficiency reaches 99.4% for more than 600 cycles in the 4% EC electrolyte. The cycle life of Zn stripping/plating in symmetric cells extends from 108 h to 1800 h at 1 mA cm^−2^ and 1 mA h cm^−2^ after the addition of EC. The cycle life also reaches 180 h with 35% DOD in 4% EC, whereas large voltage fluctuation is observed at the beginning in the ZnSO_4_ electrolyte. The Zn//V_6_O_13_·H_2_O full cell with N/P = 1.3 realizes 80.5% capacity retention after 500 cycles in 4% EC, superior to 51.1% with EC-free electrolyte. Our results show that interface regulation is an efficient way to promote the electrochemical behavior of the Zn anode. It allows the reduction of additive content so that the aqueous nature of bulk electrolyte is maintained. It would put forward new approaches for high-safety aqueous Zn batteries.

## Data availability

Data are available from the authors on reasonable request.

## Author contributions

K. W., T. Q. and X. S. conceived and designed this work. K. W. and T. Q. carried out the synthesis, electrochemical measurements and computational calculations. K. W., T. Q., L. L., F. L., J. Z. and X. L. participated in the analysis of the data. All authors discussed and revised the manuscript.

## Conflicts of interest

There are no conflicts to declare.

## Supplementary Material

SC-014-D3SC01831H-s001
