# Supplementary material for: Interface solvation regulation stabilizing the Zn metal anode in aqueous Zn batteries
Source: Chem Sci. 2023 Jul 11;14(30):8076–83. doi: 10.1039/d3sc01831h (PMC10395310; doi:10.1039/d3sc01831h)
Supplement: SC-014-D3SC01831H-s001 [file SC-014-D3SC01831H-s001.pdf]

Electronic Supplementary Material (ESI) for Chemical Science.
This journal is © The Royal Society of Chemistry 2023

Electronic Supplementary Information

Interface solvation regulation stabilizing the Zn metal anode in aqueous Zn batteries

Kuo Wang[a,b], Tong Qiu[a,b], Lu Lin[a], Fangming Liu[a], Jiaqi Zhu[a], Xiao-Xia Liu[a] and Xiaoqi Sun[a,*]

[a.] Department of Chemistry, Northeastern University, 3-11 Wenhua Road, Shenyang 110819, China.

[b.] These authors contributed equally to this work.

*Corresponding author

E-mail: sunxiaoqi@mail.neu.edu.cn

1 Experimental procedures

1.1 Preparation of $V_6O_{13}·H_2O$

$V_6O_{13}·H_2O$ was synthesized according to a previous method[1]. In a typical synthesis, 2.73 g V_2O_5 and 4.52 g $H_2C_2O_4$ were added into 40 mL deionized water. The mixture was stirred at 90 °C for 1 h and transferred into a Teflon-lined autoclave. Subsequently, 10 mL H_2O_2 and 30 mL ethanol were added. The autoclave was heated at 180 °C for 3 h. The product was filtered, washed with deionized water and ethanol, and dried at 60 °C under vacuum overnight.

1.2 Characterizations

Contact angles were measured on JY-82. Raman spectroscopy was conducted on BWS465-532S (B&W Tek Inc.) with a 532 nm excitation laser. Fourier transform infrared (FT-IR) was carried out on VERTEX70 (Bruker). X-ray photoelectron spectroscopy (XPS) was performed on Thermo Scientific K-Alpha. The data was analyzed using CasaXPS software and calibrated by referencing the C 1s peak to 284.8 eV. pH was monitored on PHS-3CB. X-ray diffraction (XRD) was performed on a PANalytical Empyrean diffractometer with Cu Kα radiation. The operando zinc deposition microscope images were recorded on metallurgical microscope (CMM-90AE). The morphologies were obtained on a SU8010 scanning electron microscope (HITACHI).

1.3 Electrochemical measurements

The EC-containing electrolytes were prepared by adding EC into 3 m $ZnSO_4$ aqueous solution. Cyclic voltammetry (CV) for electrochemical double layer capacitance (EDLC) analysis was performed in Zn//Zn Swagelok® cells. The EDLC values were obtained by the linear fits of i-v plots according to the equation of $C_{dl} = i/v$. Linear polarization was carried out with Zn foil, Cu foil and saturated calomel electrode (SCE) as the working, counter and reference electrodes, respectively. Zn^{2+} transference numbers were obtained in symmetric Zn cells. Electrochemical impedance spectroscopy (EIS) was performed at the pristine state. A voltage polarization of 20 mV was then applied until the stabilization of current, and EIS measurements were carried out again. The transference numbers were calculated according to the following equation:

$$t_+ = \frac{I_{ss}(\Delta V - I_0 R_0)}{I_0(\Delta V - I_{ss} R_{ss})}$$

ΔV was the polarization voltage. I_0 and I_{ss} were the current before and after the polarization process. R_0 and R_{ss} were the charge transfer resistances before and after

polarization process, which were obtained by fitting the Nyquist plots. Chronoamperometry (CA) measurements were performed in three-electrode cells with Zn foil as the working, counter and reference electrodes. Voltage windows were measured with Ti foil as cathode and Zn foil as anode. The electrochemical performance of Zn//Zn, Zn//Cu and Zn//$V_6O_{13}·H_2O$ cells were tested in 2032 coin-type cells. We noted that each batch of Zn altered a little in the electrochemical performance, and we made sure that the same batch was applied in this single paper for direct comparisons. The $V_6O_{13}·H_2O$ cathode was prepared by mixing the active material, Super P and polyvinylidene difluoride (PVDF) at a mass ratio of 6:3:1 in N-Methyl pyrrolidone (NMP) and drop casted on graphite foil. The Zn//$V_6O_{13}·H_2O$ cells were sealed by pressing at 500 kg cm^{-2} pressure to allow sensitive detection of different rate and cycling performance. All electrochemical measurements were performed on Bio-logic VMP3 or LANHE CT2001A battery test systems.

1.4 Computational methods

The molecular orbital energy levels and energy calculations were conducted in the Gaussian 16 program. The structure optimizations were performed at the B3LYP/6–31G(d,p) level.

The binding energies between Zn^{2+} and different solvent molecules were calculated by the following equation:

$$E_b = E_{molecules+Z\ \ (2+)} - E_{molecules} - E_{Zn(2+)}$$

E_b was binding energy between Zn^{2+} and solvent molecules. $E_{molecules+Zn(2+)}$, $E_{molecules}$ and $E_{Zn(2+)}$ were the energies of Zn^{2+}-solvent complex, solvent molecules and Zn^{2+}, respectively.

The adsorption energies of solvent molecule on Zn were calculated by the DMol3 program package in Materials Studio. The exchange and correlation terms were determined using the Generalized Gradient Approximation (GGA) in the form proposed by Perdew, Burke, and Ernzerhof (PBE). The energy convergence criterion was set to 10^{-6} Hartree. All calculations including geometry optimization, single-point energy and electronic density were carried out within a 7.99*14.84*22.69 $Å^3$ box under a periodic boundary condition. In the Z direction, there was about 15 Å vacuum for erasing the effect of periodic condition for slab model. A 4*4 supercell with four-layer Zn (100) face was used to represent the adsorbed surface, and the bottom two layers were kept fixed to maintain bulk properties.

The adsorption energies of different solvent molecules on Zn slab were calculated by the following equation:

$$E_{adsorb} = E_{molecule+Zn} - E_{molecule} - E_{Zn}$$

E_{adsorb} was the adsorption energy. $E_{molecule+Zn}$, $E_{molecule}$ and E_{Zn} were the energies of the system, solvent molecule and Zn, respectively.

The charge differential density was calculated by the following equation:

$$\Delta\rho = \Delta\rho_{molecule+Z} \;\; - \Delta\rho_{molecule} - \Delta\rho_{Zn}$$

Δρ was differential charge density of system. $\Delta\rho_{molecule+Zn}$, $\Delta\rho_{molecule}$ and $\Delta\rho_{Zn}$ were the charge density of the system, solvent molecule and Zn, respectively.

For molecular dynamics (MD) simulations, the model was constructed with 556 H_2O, 30 $ZnSO_4$ and 6 EC, which agreed with the composition in 4% EC. The COMPASSII force fields were selected for assigning charges for Zn^{2+}, SO_4^{2-}, and EC. The geometry optimization was carried out in the Forcite module, in which the lattice geometry was optimized based on the convergence of total energy (0.001 kcal mol^{-1}) with the force of 0.5 kcal $mol^{-1}Å^{-1}$. MD simulations were then conducted by NVT and NPT ensembles at 298 K. The cutoff distance for van der Waals and the electrostatic interactions were 12.5 Å and 12 Å, respectively. All simulations were carried out with the standard periodic boundary condition and the simulation time was long enough to ensure the equilibrium states of electrolyte systems. The radial distribution functions (RDFs) of different species were calculated with the following equation:

$$g(r) = \frac{\rho(r)}{\rho}$$

r was the distance from target particle to Zn^{2+}. ρ(r) was the average particle density between r and r+dr. ρ was the total average particle density.

The radius-dependent coordination numbers of different species were calculated by the following equation:

$$N(r) = 4\pi\rho \int_0^r g(r) r^2 dr$$

2. Supplementary figures, table and discussions

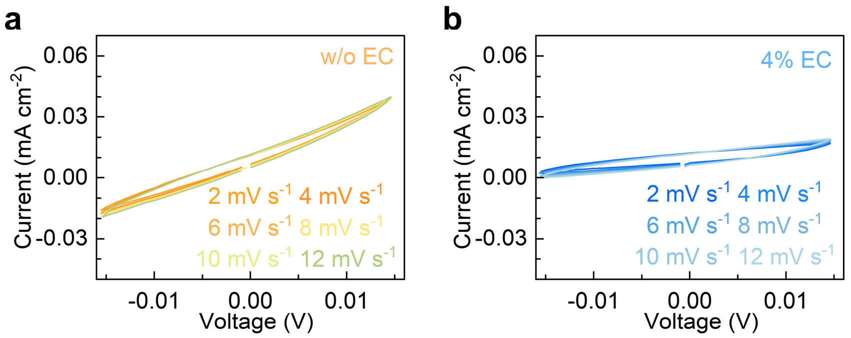

Fig. S1 CV curves of Zn//Zn symmetric cells in the non-Faradic range in (a) 3 m $ZnSO_4$ and (b) 4% EC.

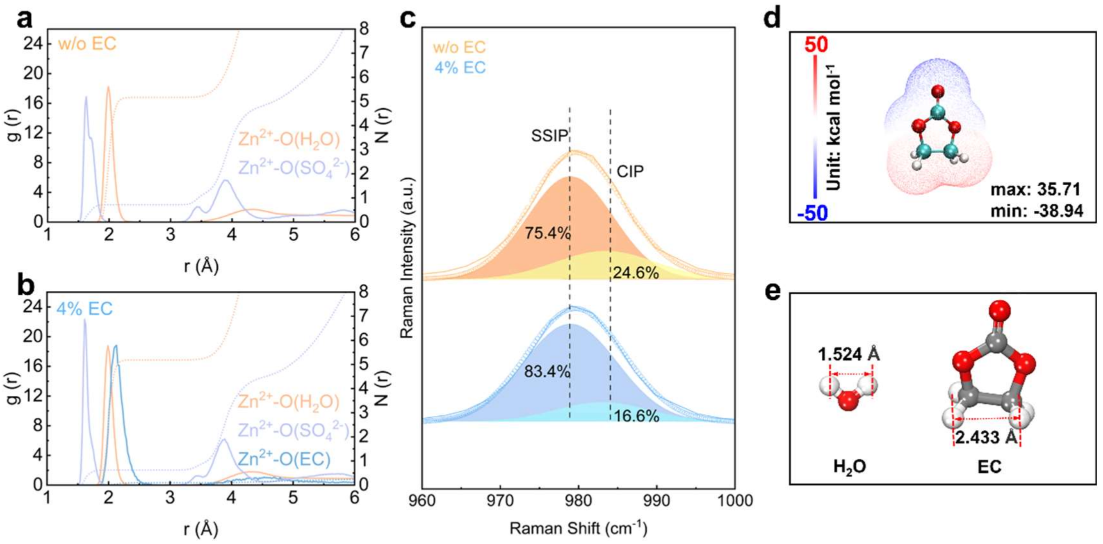

Fig. S2 The RDFs and radius-dependent coordination numbers in 3 m $ZnSO_4$ (a) without and (b) with 4% EC. (c) Raman spectra of the two solutions. (d) The electrostatic potential map of EC molecule. (e) The sizes of H_2O and EC tails.

The inner solvation shells of Zn^{2+} in the two electrolytes are examined by MD simulation and Raman analysis. Fig. S2a,b shows the RDFs. The result suggests the low participation of SO_4^{2-} in the inner solvation shell of Zn^{2+} in either system. In accordance, the contact ion pair (CIP) component with SO_4^{2-} participation obtained by Raman spectrum is determined to be only 24.6% in the $ZnSO_4$ solution, which further decreases to 16.6% after EC addition (Fig. S2c). The decreased participation of SO_4^{2-} in Zn^{2+} solvation shell is attributed to the high degree of charge separation in EC molecule, as shown in the molecular electrostatic potential map (Fig. S2d). With EC entering the inner solvation shell, the carbonyl group with negative potential coordinates with Zn^{2+}, whereas the carbon end of the ring with positive potential brings SO_4^{2-} outside of inner sheath. The large size of EC tail also provides steric hindrance (Fig. S2e). The low coordination number of EC from the calculation is due to its low overall content in the bulk electrolyte. The situation changes at the EC-rich interface. Overall, with the low participation of SO_4^{2-} in the inner solvation shell, its effect is not considered in free energy calculations.

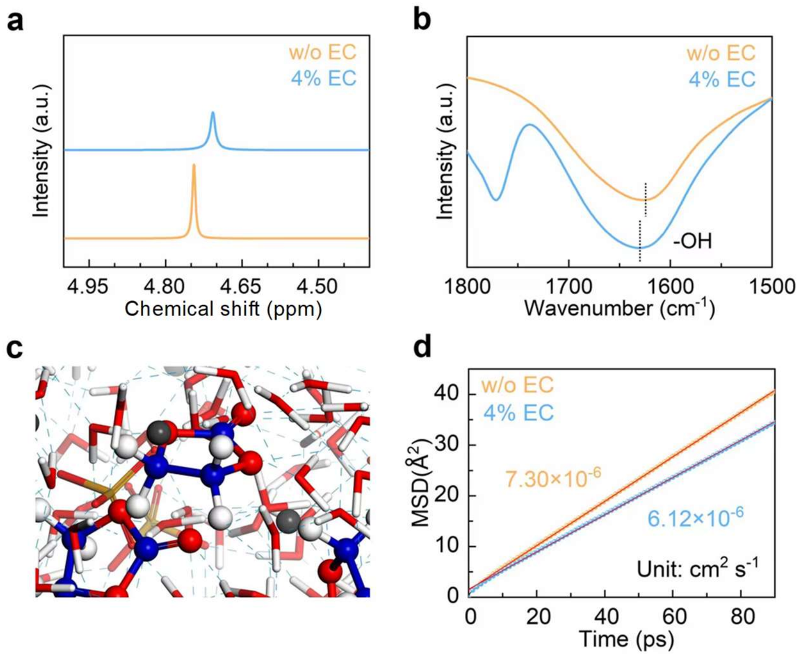

Fig. S3 (a) ¹H NMR and (b) FT-IR spectra of 3 m $ZnSO_4$ and 4% EC solutions. (c) Snapshot of H-bond network in 4% EC solution. (d) MSD plots of H_2O molecules in different solutions.

Fig S3a shows the ¹H NMR of 3 m $ZnSO_4$ and 4% EC solutions. The hydrogens on water show up at the chemical shift of 4.74 ppm in 3 m $ZnSO_4$, which experiences a down-field shift to 4.71 ppm with the introduction of 4% EC. It demonstrates the stronger shielding effect by forming stronger hydrogen bonds with EC. Fig S3b shows the FT-IR spectra. The O-H vibration experiences blue-shift after adding 4% EC, again demonstrating the hydrogen bond formation between water and EC. The hydrogen bonds among EC and H_2O molecules are also presented in the optimized MD box of 4% EC (Fig S3c). The MSD method is applied to calculate the diffusion coefficients of water in 3 m $ZnSO_4$ and 4% EC (Fig S3d). The smaller value in the later corresponds to slower water movement. It results from the interruption of original hydrogen bond network of water by EC, which helps to decrease water activity.

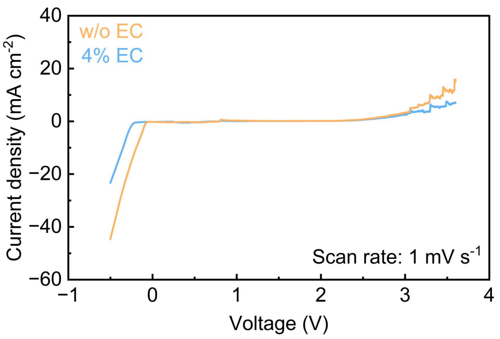

Fig. S4 Voltage windows of the two electrolytes.

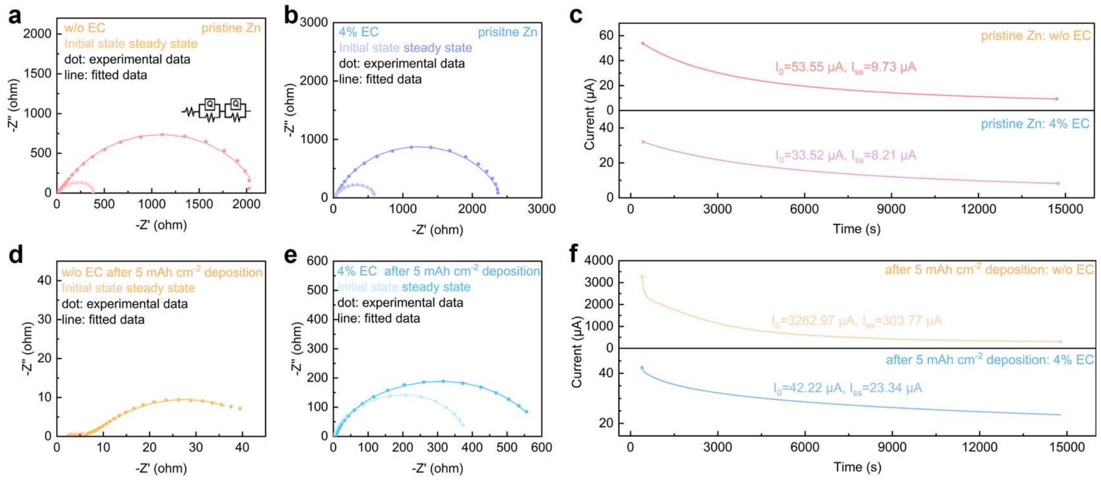

Fig. S5 Nyquist plots of (a,b) pristine Zn electrode and (d,e) after 5 mAh cm^{-2} deposition in (a,d) 3 m $ZnSO_4$ and (b,e) 4% EC electrolytes at the initial and steady states, and (c,f) current response with a polarization voltage of 20 mV.

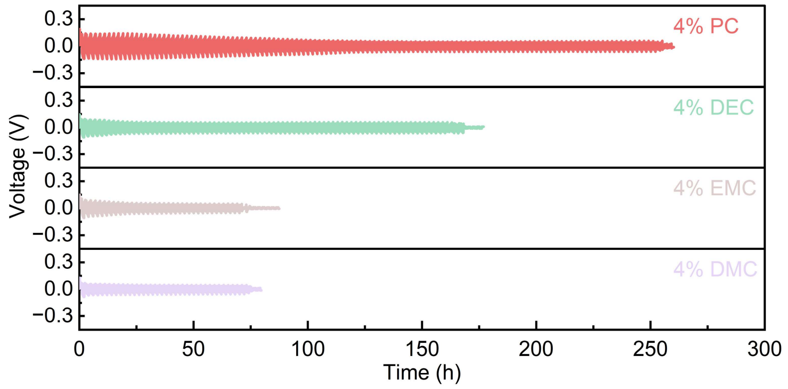

Fig. S6 The lifespans of Zn//Zn symmetric cells at 2 mA cm^{-2} and 2 mAh cm^{-2} in 3 m $ZnSO_4$ with different carbonate additives.

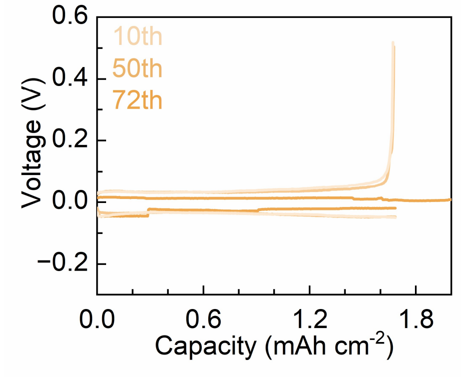

Fig. S7 Voltage curves of Zn//Cu cells at different cycles in 3 m $ZnSO_4$.

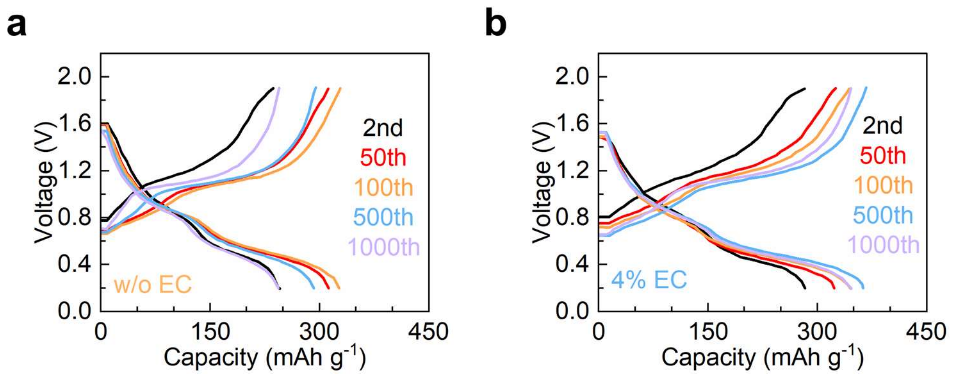

Fig. S8 Charge-discharge curves of $V_6O_{13}\cdot H_2O$ cathode with excess Zn anode and (a) 3 m $ZnSO_4$ and (b) 4% EC electrolytes.

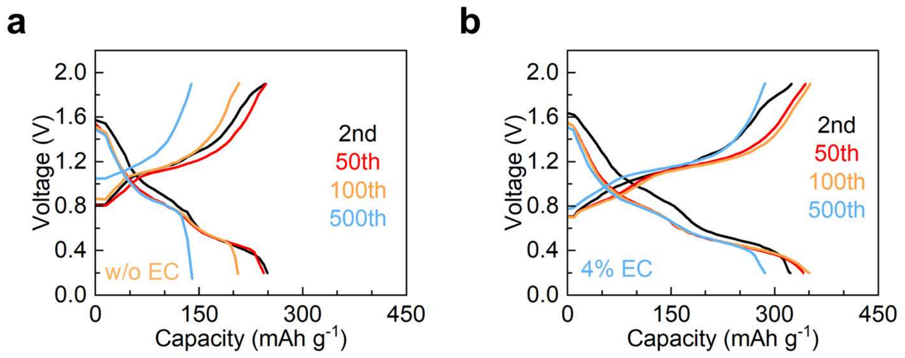

Fig. S9 Charge-discharge curves of $V_6O_{13}\cdot H_2O$ cathode with limited Zn anode of N/P = 1.3 and (a) 3 m $ZnSO_4$ and (b) 4% EC electrolytes.

Table S1. The cycling stability of zinc electrode using 4% EC electrolyte in comparison to other additives reported in previous studies.

Electrolyte additive applied	Current density ($mA\ cm^{-2}$)/ Areal capacity ($mAh\ cm^{-2}$)	Cycle life (h)	Cycle life extension comparing to the blank electrolytes	Refs.
100 mM xylitol	1/1	1100	6.1 times	[2]
0.004 M $NiSO_4$	1/1	900	4.5 times	[3]
75 mM Na_4EDTA	2/2	450	5.1 times	[4]
20 wt% DMSO	0.5/0.5	1000	2.6 times	[5]
8.5 m $La(NO_3)_3$	1/1	1200	3.8 times	[6]
10% TG	2/0.67	1000	11.1 times	[7]
4% EC	1/1	1800	16.7 times	Our work
	2/2	600	12.5 times	

3. References

1. J. Lai, H. Zhu, X. Zhu, H. Koritala and Y. Wang, Interlayer-Expanded $V_6O_{13}{\cdot}nH_2O$ Architecture Constructed for an Advanced Rechargeable Aqueous Zinc-Ion Battery, *ACS Applied Energy Materials*, 2019, **2**, 1988-1996.
2. H. Wang, W. Ye, B. Yin, K. Wang, M. S. Riaz, B.-B. Xie, Y. Zhong and Y. Hu, Modulating Cation Migration and Deposition with Xylitol Additive and Oriented Reconstruction of Hydrogen Bonds for Stable Zinc Anodes, *Angewandte Chemie International Edition*, 2023, **62**, e202218872.
3. Y. Dai, C. Zhang, W. Zhang, L. Cui, C. Ye, X. Hong, J. Li, R. Chen, W. Zong, X. Gao, J. Zhu, P. Jiang, Q. An, D. J. L. Brett, I. P. Parkin, G. He and L. Mai, Reversible Zn Metal Anodes Enabled by Trace Amounts of Underpotential Deposition Initiators, *Angewandte Chemie International Edition*, 2023, **62**, e202301192.
4. S.-J. Zhang, J. Hao, D. Luo, P.-F. Zhang, B. Zhang, K. Davey, Z. Lin and S.-Z. Qiao, Dual-Function Electrolyte Additive for Highly Reversible Zn Anode, *Advanced Energy Materials,* 2021, **11**, 2102010.
5. L. Cao, D. Li, E. Hu, J. Xu, T. Deng, L. Ma, Y. Wang, X.-Q. Yang and C. Wang, Solvation Structure Design for Aqueous Zn Metal Batteries, *Journal of the American Chemical Society,* 2020, **142**, 21404-21409.
6. R. Zhao, H. Wang, H. Du, Y. Yang, Z. Gao, L. Qie and Y. Huang, Lanthanum nitrate as aqueous electrolyte additive for favourable zinc metal electrodeposition, *Nature Communications,* 2022, **13**, 3252.
7. Z. Liu, R. Wang, Q. Ma, J. Wan, S. Zhang, L. Zhang, H. Li, Q. Luo, J. Wu, T. Zhou, J. Mao, L. Zhang, C. Zhang and Z. Guo, A Dual-Functional Organic Electrolyte Additive with Regulating Suitable Overpotential for Building Highly Reversible Aqueous Zinc Ion Batteries, *Advanced Functional Materials*, 2023, **33**, 2214538.
